# Intended reduction in formulated net energy density and associated changes in diet composition improved growth performance and breast meat yield in broiler chickens under both cyclic heat stress and thermoneutral conditions

**DOI:** 10.1016/j.psj.2026.107759

**Published:** 2026-09-12

**Authors:** Carria Xie, Peter H. Selle, Shemil P. Macelline, Mushin Al Anas, Mehdi Toghyani, Eunjoo Kim, Reza Barekatain, Sonia Y. Liu

**Affiliations:** a School of Life and Environmental Sciences, Faculty of Science, The University of Sydney, Camperdown, New South Wales, 2006, Australia; b Poultry Research Foundation, The University of Sydney, Camden, New South Wales, 2570, Australia; c Sydney School of Veterinary Science, The University of Sydney, Camden, New South Wales, 2570, Australia; d Faculty of Animal Science, Universitas Gadjah Mada, Yogyakarta, 55281, Indonesia

**Keywords:** Carcass traits, Fiber, Growth, Heat stress, Net energy

## Abstract

This experiment investigated the effects of diets formulated to two net energy densities (2360 versus 2260 kcal/kg) and the impacts of cyclic heat-stress on the performance of broiler chickens via a 2 × 2 factorial array of treatments. Cyclic heat-stress consisted of the daily exposure of birds to an environmental temperature of 33°C for 8 hours as opposed to thermo-neutral conditions. Parameters assessed included growth performance from 14 to 35 days post-hatch, carcass traits, breast-meat myopathies, jejunal and ileal digestibility coefficients of starch, gross energy and protein (N). Cyclic heat stress significantly compromised weight gain, feed intake and FCR but did not influence breast-meat yields and myopathies, relative fat pad weights or jejunal and ileal digestibility coefficients for starch, protein and gross energy. The reduction in formulated dietary net energy improved FCR by 2.07% (*P* = 0.018) and increased breast-meat yields by 11.3% (*P* = 0.003). Millrun was included in the diets at 109 g/kg, and dietary crude protein was reduced, non-bound amino acids were included to achieve the intended reduction in formulated NE density. As this reduction in NE was achieved through concurrent changes in diet composition, the observed responses cannot be attributed to dietary NE alone. Importantly, these dietary modifications are inherent to practical NE-based feed formulation strategies and highlight the need for a greater understanding of the roles of dietary fibre or non-starch polysaccharides in influencing growth performance and carcass traits in broiler chickens.

## Introduction

Over 20 years ago, heat-stress was estimated to generate a US $165 million loss across the United States poultry industries indicating the need for more energy and capital efficient heat abatement systems (St-Pierre et al., 2003). Heat stress is one of the most important environmental stressors challenging global chicken-meat production and yet our knowledge of the underlying mechanisms driving the reported impacts of heat-stress is incomplete (Lara and Rostagno, 2013) . Moreover, this complex problem will be only exacerbated by global warming. Indeed, climate change is a very real challenge to continuing, sustainable chicken-meat production as sub-optimal environmental conditions will prevent birds from realising their extraordinary genetic potential for rapid growth (Oke et al., 2024). The negative impacts of heat stress on the growth performance of broiler chickens were given detailed consideration in two related reviews by Brugaletta et al. (2022) and Teyssier et al. (2022). The first review provided insights into physiology and gut health, and the second, insights into the utilisation of protein and energy. While broiler chickens are homoeothermic; nevertheless, they are highly susceptible to heat-stress because they cannot dissipate heat efficiently due to the lack of sweat glands (Mangan and Siwek, 2024). In essence, broiler chickens exposed to heat stress suppress their feed intakes to lower metabolism and regulate body temperature, which compromises growth performance and the profitability of production (Oluwagbenga and Fraley, 2023).

Dietary energy density is an expensive component in chicken-meat production and yet the correct quantification of energy remains elusive (Chrystal et al., 2022). In poultry, both apparent metabolisable energy (**AME**) and nitrogen corrected metabolisable energy values have been used for feed formulation for decades, but their accuracy is inconsistent. Indeed, Wu et al. (2020) have reviewed the “historical flaws” in bioassays used to generate metabolisable energy values for the formulation of poultry diets. Nitrogen-corrected apparent metabolisable energy (**AMEn**) may be calculated by correcting nitrogen retention to zero using the factor of 36.54 kJ/g N retained in the body, which is based on the data of Hill and Anderson (1958). However, Abdollahi et al. (2021) compared AME with AMEn to determine the metabolisable energy of four protein sources: soybean meal, full-fat soybean, rapeseed meal and maize distiller’s dried grains with solubles. The researchers concluded that correcting AME values to zero nitrogen retention for modern broilers penalises the energy value of protein sources and the penalty increases with the quality of the protein source. The Abdollahi et al. (2021) adjudication is at odds with the Lopez and Leeson (2007) assessment that nitrogen correction is warranted and AMEn values are feasible.

A more recent approach for poultry is the net energy (**NE**) system which is well accepted by swine nutritionists (van der Klis and Jansman, 2019). Essentially, one way to describe NE is that it is an adjustment to AME by subtracting the heat increment generated by the digestion, assimilation and metabolism of feedstuffs. Fasting heat production is indicative of heat increment and the fasting heat production per kg bodyweight (BW^0.70^) in broiler chickens at zero activity and under thermoneutral conditions ranged between 420 and 450 kJ/day in Noblet et al. (2015).

In broad terms, protein meals generate the highest heat increment, feed grains are intermediate, and lipids (fats and/or oils) generate the lowest heat increment. The topic of heat increment in poultry is considered in more detail in Musharaf and Latshaw (1999). The wider acceptance of the NE system for pigs than broiler chickens may stem from the dietary fiber component. Pig diets contain higher levels of crude fiber than poultry diet and fiber is more completely digested in pigs because of their greater hindgut fermentation capacity (van der Klis and Jansman, 2019). Thus, the allowance for neutral detergent fiber (**NDF**) in the NE equation would carry more importance in pigs than poultry. Nevertheless, the NE proponents, including Noblet et al. (2024), contend that the NE system provides more accurate representations of dietary energy and may be more accurately predict broiler performance than the AME system. Moreover, there are indications (Chang et al., 2016; Sharma et al., 2018) that the optimum dietary metabolisable energy density is now lower than levels being recommended in practice. This is reflected by the recent downward revisions of recommended dietary energy densities by the two largest broiler breeders (Toghyani et al., 2024). Presently, one recommendation for broiler chickens with a target live weight of 2.0 to 3.5 kg liveweight ranges from 12.4 for 1 to 10 days, to 12.8 for 11 to 24 days and 13.0 MJ/kg for 25 to 39 days post-hatch (Aviagen, 2022).

Evaluating dietary energy impact on animal performance is inherently challenging because dietary energy cannot be altered in isolation. Even in studies focused on a single ingredient or nutrient, changes to other dietary components are often necessary to form isoenergetic diets with balanced amino acid profiles. Therefore, it is our view that energy studies should not be viewed as single-ingredient or single-nutrient studies. From a practical nutritionist perspective, implementation of NE systems affects multiple dietary characteristics simultaneously through least-cost formulation, including crude protein, fat and fiber or NDF concentrations. Consequently, the present study compared practical diets formulated to two estimated NE densities rather than manipulating a single ingredient or nutrient. This approach better reflects the fact that reductions in dietary crude protein and increased use of lower-cost by-product ingredients are major incentives for adoption of NE systems. Although this design limits the ability to isolate the effects of individual dietary components, it allows a more realistic evaluation of the overall outcomes associated with practical NE-based feed formulation. The present study adopted an end-user perspective and evaluated broiler responses to diets formulated to different predicted NE densities and the aim was not to validate the prediction equation itself.

Given that the heat increment associated with the digestion and metabolism of crude protein and fiber is explicitly accounted for in the NE system, the potential advantages of NE-based diet formulation under heat-stress conditions warrant investigation. Therefore, the objective of the present study was to determine the effects of diets containing two estimated NE densities (2360 vs. 2260 kcal/kg) and cyclic heat stress on the performance of broiler chickens from 14 to 35 days post-hatch in a 2 × 2 factorial arrangement of treatments. As NE has been proposed to more closely reflect the energy available to support growth and production, it was hypothesised that the high-NE diet would improve growth performance and feed efficiency, especially under cyclic heat stress conditions. However, recent evidence suggests that the optimum dietary energy density for modern broilers may be lower than current breeder recommendations (Toghyani et al., 2024). Therefore, a secondary hypothesis was that an intended reduction in formulated NE density would not compromise growth performance while enabling greater use of by-product ingredients in feed formulation.

In addition to growth performance, dietary energy and nutrient composition may influence carcass composition and breast meat quality. Breast muscle myopathies, including woody breast and white striping, are generally associated with rapid muscle growth and high breast meat yield (Toghyani et al., 2025). The dietary treatments evaluated in the present study differed not only in estimated NE density but also in associated crude protein, ether extract and fiber concentrations. Furthermore, heat stress is known to alter nutrient utilisation, energy metabolism and oxidative status, which may further affect breast meat quality (Kuttappan et al., 2021). Hence, indicators of breast meat quality were also evaluated in the present study.

## Materials and methods

### Ethics approval

The conduct of this study fully complied with the specific experimental protocol (2025/AE 000080) approved by the Research Integrity and Ethics Administration of The University of Sydney.

### Experimental design

This experiment was based on a 2 × 2 factorial array of treatments, which were applied from 14 to 35 days post-hatch to broiler chickens. The two intended dietary NE densities were 2260 kcal/kg and 2360 kcal/kg or a difference of 100 kcal/kg. Birds were held under heat-stress versus thermo-neutral conditions from 25 to 35 days post-hatch, where cyclic heat-stress consisted of exposure to a temperature of 33°C for 8 hours per day.

### Bird management

A total of 280 day-old, off-sex, male Ross 308 birds were procured from a commercial hatchery (Aviagen Hatchery, 9 Tait Cres, Goulburn NSW 2580). Upon arrival, birds were placed into a floor pen facility mimicking commercial production. A common commercial diet was fed to all birds from 1 to 13 days post hatch. As pen litter bedding material, clean wood shavings were placed in pens prior to bird allocation. Water and feed were offered *ad libitum* for the duration of the feeding trial. A lighting program of 23 h light and 1 h dark was used during the first week and gradually adjusted to 16 h light and 8 h dark from the end of the first week until the end of the experiment (d 35 post-hatch). An initial room temperature of 32 ± 1°C was maintained for the first week, then gradually decreased to 22 ± 1°C by day 25, and maintained at this temperature for the duration of the feeding trial for the thermoneutral treatment birds.

On day 14, birds were reassigned into their respective treatments and distributed into 40 floor pens with 10 replicated pens per treatments and 7 birds per replicate. Dimensions of the floor pens were 0.57 m in width and 0.98 m in length. Cyclic heat stress was applied to half of the birds from day 25 to day 35 post hatch. Birds assigned to the cyclic heat stress treatment were exposed to 31°C continuously for 8 h daily (09:00 to 17:00 h) and maintained at 22°C for the remaining 16 h until the end of the experimental period. Measurement temperature and humidity in the four experimental rooms during the heat-stress period was shown in Figure 1.

**Figure 1:**
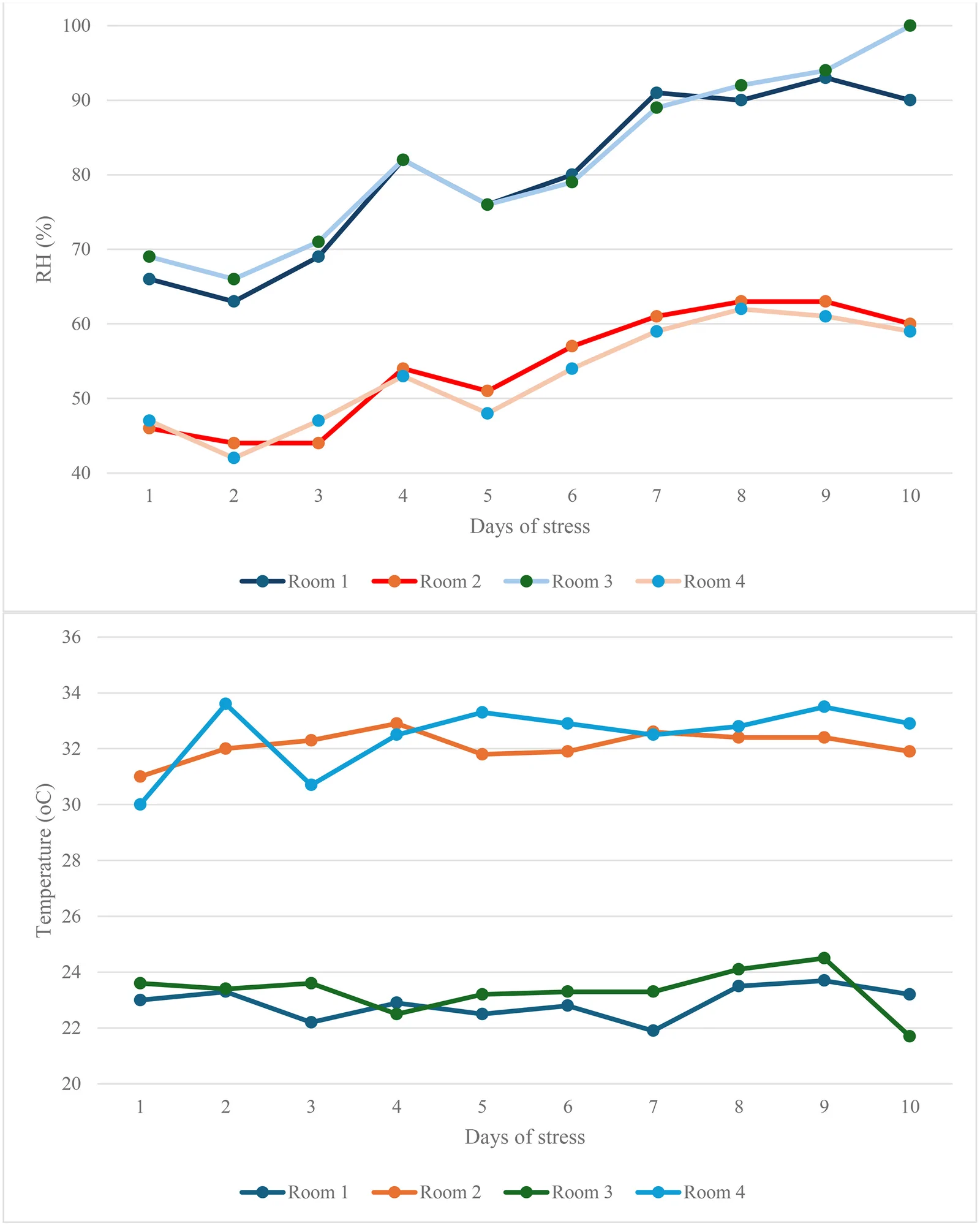
Afternoon temperature and humidity in the four experimental rooms during the heat-stress period. Measurements were taken daily between 3:00 and 4:00 pm.

### Diet preparation

Prior to diet formulation, representative subsamples of wheat, soybean meal, meat and bone meal, canola meal and canola seed were analyzed by near-infrared spectroscopy to predict proximate analysis, digestible amino acid concentrations, and AME using AMINONIR®PROX, AMINONIR®NIR, and AMINONIR® NRG (Evonik Nutrition & Care, Hanau Germany), respectively. Diets were based mainly on wheat (110 g/kg CP; AME 3,180 kcal/kg AME) and soybean meal (470 g/kg CP; 2,400 kcal/kg AME) as shown in Table 1 and Table 2.

**Table 1:** Composition of experimental diets.

Feed ingredient (g/kg)	Common starter diet (1 to 13 days)	Dietary treatments (14 to 35 days)	
		2360 kcal/kg NE	2260 kcal/kg NE
Wheat (11%)^b^	626	651	593
Soybean meal (47.0%)	264	208	151
Millrun (15.0%)	-	-	108.5
Canola meal (36.5%)	30.0	40.0	40.0
Canola seed	30.0	40.0	40.0
Canola oil	7.50	26.0	24.5
Limestone	12.3	10.9	11.0
Dicalcium phosphate	8.88	3.24	3.62
Sodium chloride	1.46	1.35	1.03
Sodium bicarbonate	3.65	3.35	4.30
Lysine HCl	4.50	3.35	4.85
DL-methionine	3.75	2.90	3.35
L-threonine	2.10	1.35	2.05
L-arginine	1.15	0.60	1.83
L-isoleucine	0.58	0.24	1.19
L-leucine	-	-	0.57
L-valine	1.0	0.41	1.25
Phytase	0.15	0.15	0.15
Xylanase	0.15	0.15	0.15
Titanium dioxide	-	5.0	5.0
Vitamin-mineral premix^a^	2.0	2.0	2.0

a The vitamin-mineral premix contained (per kg of diet): [IU] retinol, 12,000; cholecalciferol, 5,000; [mg] tocopherol, 50; menadione, 3; thiamine, 3; riboflavin, 9; pyridoxine, 5; cobalamin, 0.025; niacin, 50; pantothenate, 10; folate, 2; biotin, 0.2; copper, 20; iron, 40; manganese, 110; cobalt, 0.25; iodine, 1; molybdenum, 2; zinc, 90; selenium, 0.3.

b Values presented in parentheses indicate protein content.

**Table 2:** Nutrient specifications of experimental diets.

Formulated Specification (g/kg)	Common starter diet	Dietary treatments (14 to 35 days)	
		2360 kcal/kg NE^a^	2260 kcal/kg NE
AME (kcal/kg)	2975	3091	2985
NE (kcal/kg)^a^	2242	2360	2260
Crude protein	223	201	191
Lysine^b^	13.2	11.2	11.2
Methionine	6.60	5.50	5.80
Methionine + cysteine	10.0	8.80	8.80
Threonine	8.80	7.50	7.50
Isoleucine	8.80	7.70	7.70
Leucine	14.6	12.1	12.1
Tryptophan	2.60	2.30	2.10
Arginine	14.0	12.1	12.1
Histidine	5.00	4.50	4.10
Valine	10.0	8.70	8.70
Crude fat	36.2	58.3	58.0
Crude fiber	31.2	32.3	39.1
Starch	393	311	297
Calcium	9.50	8.00	8.00
Available phosphorus	5.00	4.00	4.00
Dietary electrolyte balance (mEq/kg)	223	208	203
*Analysed nutrients*			
Gross energy (kcal/kg)		4099	4122
Crude protein (N × 6.25)		198.0	194.7
Fat by acid hydrolysis		64	69
NDF		101	127
Calculated NE based on analysed nutrients		2369	2272

a NE calculated as: AME × 0.815 – 0.026 × crude protein + 0.020 × ether extract –0.024 × NDF. Noblet et al. (2024).

b Amino acid concentrations expressed on a digestible basis.

Dietary NE densities (MJ/kg) were calculated by the following equation developed by Noblet et al. (2024):

AME × 0.815 – 0.026 × crude protein + 0.020 × ether extract – 0.024 × NDF

The diets were balanced for the essential amino acids (lysine, total sulphur amino acids, threonine, tryptophan, arginine, isoleucine, valine) by following the ideal amino acids ratios recommended by (Aviagen, 2022). Diets were initially formulated to meet breeder recommendations using AME as the primary energy system. The baseline NE concentrations of the diet were subsequently estimated using the NE prediction equation and the baseline diet were reformulated based on the calculated NE without fixing AME density. To generate the low-NE treatment, diets were then formulated to reduce estimated NE by approximately 100 kcal/kg relative to the baseline diet. During this process, AME was also allowed to vary, as maintaining a constant AME while achieving the targeted NE reduction was not practically feasible using the selected NE equation. All diets were supplemented with both phytase (Axtra® PHY GOLD 10T phytase 1500 FTU/kg) and xylanase (Ronozyme® WX 2000) at 150 g/tonne. The phytase matrix was based on the manufacturer’s recommendations for minerals at 1500 FTU/kg of the diet. The matrix values applied were 0.195% for available P, 0.207% for Ca, and 0.035% for Na. No matrix values for energy or amino acids were applied for phytase. For xylanase, an energy matrix was applied only to wheat and millrun, with their ME values increased by 3.5%. Following application of the xylanase matrix, the AME values used in formulation were 3,180 kcal/kg for wheat and 2,815 kcal/kg for millrun. Millrun was analysed to contain 182 g/kg CP, 55 g/kg fat and 394 g/kg of NDF. Titanium dioxide was included across all diets at 5 g/kg as a dietary marker to determine protein, gross energy and starch digestibility coefficients. Diets were steam-pelleted at 80°C through a Palmer PP330 pellet press (Palmer Milling Engineering, Griffith NSW, Australia) with a conditioner residence time of 14 seconds.

### Data, sample collection and chemical analysis

Growth performance (weight gain, feed intake, FCR, mortality) was determined from 14 to 35 days post-hatch. The weights of any dead or culled birds were recorded daily to correct feed intakes and FCR calculations. The birds were euthanized via intravenous injections of sodium pentobarbitone on day 35. Digesta from the distal jejunum and distal ileum were collected and both samples were pooled by pen, homogenized, freeze-dried and ground through a 0.5 mm screen. Distal jejunum and distal ileum digesta were analysed for titanium dioxide, protein (N), gross energy and starch. Protein contents (N × 6.25) of diets and digesta were determined using a nitrogen determinator (Leco Corporation, St Joseph, MI). Gross energy (GE) of diets and digesta were determined using an adiabatic bomb calorimeter (Parr Instrument Company. Moline, IL). Starch concentrations were determined by methodology outlined in Mahasukhonthachat et al. (2010). Involving a procedure with dimethyl sulfoxide, α-amylase and amyloglucosidase. Also on day 35, 4 birds closest to the median BW were selected from each pen to determine breast-meat (*Pectoralis major*) yields and relative weights of abdominal fat-pads. Additionally, *Pectoralis major* muscles were visually examined and scored for the occurrence of woody breast and white striping according to the methodology of Toghyani et al. (2025).

Analyses of titanium dioxide were completed following the method of Short et al. (1996). The apparent ileal and jejunal nutrient digestibility coefficients were calculated based on the following equation:

$$Digestibilitycoefficient=\frac{\left(\frac{Nutrient_{Diet}}{TiO2_{Diet}}\right)-\left(\frac{Nutrient_{Digesta}}{TiO2_{Digesta}}\right)}{\left(\frac{Nutrient_{Diet}}{TiO2_{Diet}}\right)}$$

Apparent disappearance rates of starch and protein (N) in the jejunum and ileum were calculated from the following equation:

Disappearance rate (g/bird/day) = nutrient dietary concentration (g/kg) × daily feed intake (g/bird) × nutrient digestibility coefficient.

### Statistical analysis

Normality of the data was assessed using the Shapiro-Wilk test with *P* > 0.05 indicating a normal distribution. Data collected were checked for normality prior to conducting statistical analysis. Each pen was considered as an experimental unit and values presented in the tables are treatment means with pooled standard error of mean (SEM). Data were analysed using a two-way ANOVA according to the model:

$Y_{ijk}=\mu +E_{i}+N_{j}+{\left(EN\right)}_{ij}+\varepsilon_{ijk}$

where $E_{i}$ represents the effect of environment, $N_{j}$ represents the effect of dietary net energy density, ${\left(EN\right)}_{ij}$the interaction between environment and NE density, and $\varepsilon_{ijk}$the random error.

Statistical analyses were carried out by using JMP Student Edition (SAS Institute Inc., JMP Software, Cary, NC). If a significant treatment effect was detected, differences between treatments or main effects were separated using the Tukey HSD test. Differences were considered significant at *P* < 0.05.

## Results

The effects of treatments on growth performance are shown in Table 3. Exposure to elevated temperatures compromised weight gain by 6.89% (1958 versus 2103 g/bird; *P* < 0.001), feed intake by 4.94% (2557 versus 2690 g/bird; *P* < 0.001) and FCR by 2.11% (1.307 versus 1.280; *P* = 0.017) from 14 to 35 days post-hatch. Reducing the net energy density of the diet enhanced FCR by 2.07% (1.280 versus 1.307; *P* = 0.018); however, treatment interaction was not statistically significant for weight gain (P = 0.554) or feed intake (*P* = 0.543). The overall mortality rate was 3.93%, but this was not related to treatment (*P* = 0.762).

**Table 3:** Effects of dietary treatments on growth performance of broiler chickens from 14 to 35 days post-hatch.

Treatment		Weight gain (g/bird)	Feed intake (g/bird)	FCR (g/g)	Mortality (%)
Net energy	Temperature				
2360 kcal/kg	Neutral	2092	2689	1.286	5.72
	High	1935	2569	1.328	2.86
2260 kcal/kg	Neutral	2114	2692	1.274	4.29
	High	1980	2545	1.286	2.86
SEM		20.1	21.4	0.0108	2.347
Main effects: Net energy					
2360 kcal/kg		2013	2629	1.307^b^	4.26
2260 kcal/kg		2047	2619	1.280^a^	3.58
Temperature					
Neutral		2103^b^	2690^b^	1.280^a^	5.01
High		1958^a^	2557^a^	1.307^b^	2.86
Significance (P =)					
Net energy		0.101	0.641	0.018	0.762
Temperature		< 0.001	< 0.001	0.017	0.367
Treatment interaction		0.554	0.543	0.168	0.762

a,b Means within columns not sharing a common suffix are significantly different at the 5% level of probability.

Each treatment mean represents 10 replications with 7 birds per pen.

The effects of treatments on breast-meat yields, abdominal fat-pad weights and incidences of woody breast and white striping are shown in Table 4. As a main effect, reducing the net energy density of the diets enhanced breast-meat yields by 11.3% (168 versus 151 g/kg; *P* = 0.003). Relative fat-pad weights (*P* = 0.168) and scores for white striping (*P* = 0.223) were not influenced by dietary treatments.

**Table 4:** Effects of dietary treatments on breast-meat yield (*Pectoralis major*), relative abdominal fat-pad weights, woody breast scores and white striping scores at 35 days post-hatch.

Treatment		Breast-meat yield (g/kg BW)	Fat-pad weights (g/kg BW)	Woody breast scores	White striping scores
Net energy	Temperature				
2360 kcal/kg	Neutral	153	9.88	0.93	0.48
	High	150	10.04	0.35	0.35
2260 kcal/kg	Neutral	172	9.96	0.56	0.42
	High	163	9.12	0.60	0.50
SEM		5.1	0.356	0.09	0.08
Main effects: Net energy					
2360 kcal/kg		151^b^	9.96	0.49	0.41
2260 kcal/kg		168^a^	9.54	0.58	0.46
Temperature					
Neutral		163	9.92	0.59	0.45
High		157	9.58	0.48	0.43
Significance (P =)					
Net energy		0.003	0.246	0.302	0.589
Temperature		0.260	0.340	0.191	0.806
Treatment interaction		0.641	0.168	0.079	0.223

a,b Means within columns not sharing a common suffix are significantly different at the 5% level of probability.

Each treatment mean represents 10 replications with 4 birds per pen.

The treatment outcomes for apparent digestibility coefficients of protein (N), gross energy and starch in the distal jejunum and distal ileum are shown in Table 5. Protein (N) digestibility coefficients were not influenced by treatment in the distal jejunum (*P* = 0.614) or the distal ileum (*P* = 0.365). The mean protein (N) digestibility coefficient in the jejunum was 0.733 increasing to 0.844 in the ileum. Predictably, reducing dietary net energy densities depressed gross energy digestibility by 4.72% (0.626 versus 0.657; *P* = 0.014) in the jejunum and by 3.00% (0.744 versus 0.767; *P* = 0.003) in the ileum. Also, elevated temperatures tended to depress energy digestibility by 2.23% (0.748 versus 0.763; *P* = 0.055) in the ileum. The mean starch digestibility coefficient in the jejunum was 0.883, which increased to 0.942 in the ileum. However, dietary treatments did not influence starch digestibility coefficients in the jejunum (*P* = 0.587) or ileum (*P* = 0.667).

**Table 5:** Effects of dietary treatments on apparent digestibility coefficients of protein (N), gross energy and starch in the distal jejunum and distal ileum.

Treatment			Distal jejunum			Distal ileum		
Net energy	Temperature	Protein (N)		Gross energy	Starch	Protein (N)	Gross energy	Starch
2360 kcal/kg	Neutral	0.739		0.670	0.889	0.845	0.770	0.938
	High	0.738		0.643	0.884	0.844	0.764	0.951
2260 kcal/kg	Neutral	0.733		0.633	0.875	0.842	0.755	0.929
	High	0.720		0.619	0.884	0.843	0.732	0.950
SEM		0.0116		0.0117	0.0130	0.0043	0.0072	0.0104
Main effects: Net energy								
2360 kcal/kg		0.739		0.657^b^	0.886	0.844	0.767^b^	0.945
2260 kcal/kg		0.727		0.626^a^	0.879	0.838	0.744^a^	0.940
Temperature								
Neutral		0.736		0.652	0.882	0.843	0.763	0.933
High		0.729		0.631	0.884	0.839	0.748	0.951
Significance (P =)								
Net energy		0.306		0.014	0.598	0.137	0.003	0.622
Temperature		0.551		0.089	0.911	0.348	0.055	0.102
Treatment interaction		0.614		0.587	0.587	0.365	0.232	0.667

a,b Means within columns not sharing a common suffix are significantly different at the 5% level of probability.

Each treatment mean represents 10 replications with 7 birds per pen.

The effects of treatments on starch and protein (N) jejunal and ileal disappearance rates and starch-protein disappearance rate ratios in both segments are shown in Table 6. Reducing dietary net energy depressed starch disappearance rates in the jejunum by 5.78% (32.6 versus 34.6 g/bird/day; *P* = 0.002) and in the ileum by 5.43% (34.8 versus 36.8 g/bird/day; *P* < 0.001). Increasing environmental temperature retarded jejunal starch disappearance rates by 4.94% (32.7 versus 34.4 g/bird/day; P = 0.008) and ileal disappearance rates by 3.30% (35.2 versus 36.4 g/bird/day; *P* = 0.033). Similarly, reducing dietary net energy depressed protein (N) disappearance rates in the jejunum by 3.83% (17.6 versus 18.3 g/bird/day; *P* = 0.039) and in the ileum by 2.39% (20.4 versus 20.9 g/bird/day; P < 0.015). Increasing environmental temperature retarded jejunal protein (N) disappearance rates by 5.95% (17.4 versus 18.5 g/bird/day; P = 0.002) and ileal disappearance rates by 5.12% (20.1 versus 21.2 g/bird/day; P < 0.001). Jejunal starch-protein disappearance rate ratios were not influenced (*P* = 0.215). However, in the distal ileum, reducing dietary net energy condensed starch-protein disappearance rate ratios by 2.84% (1.71 versus 1.76; *P* = 0.010). Also, increasing environmental temperature expanded starch-protein disappearance rate ratios by 2.34% (1.75 versus 1.71; *P* = 0.023) in the distal ileum.

**Table 6:** Effects of dietary treatments on starch and protein (N) disappearance rates in the jejunum and ileum (g/bird/day) and starch-protein disappearance rate ratios in the distal jejunum and distal ileum.

Treatment			Distal jejunum				Distal ileum		
Net energy	Temperature	Starch		Protein	Ratio	Starch		Protein	Ratio
2360 kcal/kg	Neutral	35.4		18.6	1.89	37.4		21.4	1.75
	High	33.6		17.9	1.88	36.2		20.4	1.77
2260 kcal/kg	Neutral	33.3		18.3	1.82	35.4		21.0	1.68
	High	31.8		17.0	1.88	34.2		19.7	1.74
SEM		0.59		0.32	0.026	0.53		0.29	0.017
Main effects: Net energy									
2360 kcal/kg		34.6^b^		18.3^b^	1.89	36.8^b^		20.9^b^	1.76^b^
2260 kcal/kg		32.6^a^		17.6^a^	1.85	34.8^a^		20.4^a^	1.71^a^
Temperature									
Neutral		34.4^b^		18.5^b^	1.86	36.4^b^		21.2^b^	1.71^a^
High		32.7^a^		17.4^a^	1.88	35.2^a^		20.1^a^	1.75^b^
Significance (P =)									
Net energy		0.002		0.039	0.145	< 0.001		0.015	0.010
Temperature		0.008		0.002	0.406	0.033		< 0.001	0.023
Treatment interaction		0.883		0.467	0.215	1.000		0.474	0.350

a,b Means within columns not sharing a common suffix are significantly different at the 5% level of probability.

Each treatment mean represents 10 replications with 7 birds per pen.

## Discussion

### General growth performance and heat stress impact

The overall mortality rate in the present study was 3.93%, but this was not related to treatment (*P* = 0.367). It is worth noting that the relatively small number of birds per replicate pen meant that mortality percentages were highly sensitive to the loss of individual birds, thereby reducing the statistical power to detect meaningful treatment effects on mortality. The average growth performance of birds kept under thermoneutral conditions in the present experiment comfortably exceeded Aviagen performance objectives for male Ross 308 broiler chickens from 14 to 35 days post-hatch (Aviagen, 2022). Weight gain exceeded objectives by 10.7% (2103 versus 1900 g/bird), while feed intake was lower by 5.68% (2690 versus 2852 g/bird), resulting in an FCR improvement of 14.7% (1.280 versus 1.501). However, cyclic heat stress significantly compromised weight gain by 6.89%, feed intake by 4.94% and FCR by 2.11% in this study. These outcomes are consistent with the meta-analysis completed by Liu et al. (2020) in which heat stress negatively impacted weight gain, feed intake and FCR across numerous comparisons to significant extents.

### Impact of intended net energy density change

The primary hypothesis was not supported, as the high-NE diet did not improve growth performance or feed efficiency. In contrast, the secondary hypothesis was supported, as the intended reduction in formulated NE density did not compromise growth performance and unexpectedly improved feed efficiency and breast meat yield under both environmental conditions. Ideally, dietary AME would remain constant while NE density varied to isolate the effects of NE. However, this is difficult to achieve using the current prediction equation because NE and AME are mathematically linked through small coefficient values of dietary crude protein, ether extract, and NDF concentrations. Based on the NE prediction equation used in the present study, a 1 percentage unit reduction in dietary CP and NDF would increase predicted NE by approximately 62 and 43 kcal/kg, respectively, whereas a 1 percentage unit increase in EE would increase predicted NE by approximately 36 kcal/kg. Therefore, achieving a 100 kcal/kg difference in NE while maintaining a constant AME concentration as intended in the present study is not readily feasible under practical least-cost feed formulation conditions. This represents a limitation of the present study, and the dietary differences associated with the intended reduction in NE should be interpreted with caution, as they do not reflect a reduction in NE per se. Rather, changes in NE were accompanied by changes in dietary composition and AME values. The following sections discuss the consequences of these dietary composition changes and their potential contribution to the observed responses.

As a main effect, reducing formulated dietary NE significantly improved FCR by 2.07% (1.280 versus 1.307), numerically increased weight gain by 1.69% (2047 versus 2013 g/bird) and fractionally depressed feed intake by 0.38%. In addition, reducing dietary NE significantly increased breast meat yield by 11.3% (168 versus 151 g/kg) as a main effect. These outcomes in the present experiment in which energy densities were assessed by net energy are not in agreement with the results reported in the below studies where energy densities were assessed by metabolisable energy.

The tolerance of broiler chickens to heat stress at two levels of dietary energy density was evaluated by Attia and Hassan (2017). Chickens were fed diets containing 220 g/kg CP with energy densities of either 3298 or 3155 kcal/kg from 28 to 49 days post-hatch under heat stress conditions, representing an energy reduction of 140 kcal/kg. The energy reduction was mainly achieved by lowering dietary inclusions of vegetable oils from 75 to 45 g/kg and heat stress consisted of exposure to 36°C for 6 hours for 4 consecutive days of the week. The energy density reduction significantly depressed weight gain by 8.75% (939 versus 1029 g/bird) and numerically compromised feed intake by 2.58% (2042 versus 2096 g/bird) and FCR by 5.85% (2.17 versus 2.05). Interestingly, the lower energy density diet accelerated respiration rates of the broiler chickens by 11.9% (76.9 versus 68.7 breaths per minute). In addition, the lower energy density diet increased uric acid plasma concentrations by 13.4% (2.80 versus 2.47 µg/mL), which is indicative of compromised protein/amino acid utilisation (Donsbough et al., 2010).

An increase in dietary metabolisable energy from 3150 to 3250 kcal/kg in association with increased inclusions of non-bound amino acids from 4.5 to 11.7 g/kg enhanced production performance and antioxidant capacity in heat stressed yellow-feathered broilers in Mao et al. (2024). The increase in dietary metabolisable energy was generated by the dietary inclusion of 17 g/kg soy oil. These dietary modifications significantly enhanced daily weight gain by 26.9% (47.6 versus 37.5 g/bird), daily feed intake by 9.32% (147.8 versus 135.2 g/bird), and FCR by 13.6% (3.12 versus 3.61) from 28 to 56 post-hatch.

Thus, the growth performance outcomes in the present experiment in which energy densities were assessed by net energy are not in agreement with the results reported in the above studies where energy densities were assessed by metabolisable energy. In these experiments, decreases in dietary metabolisable energy in diets offered to heat stressed birds compromised performance; whereas, in the present study, a decrease in net energy significantly reduced FCR and breast-meat yield.

### NE-related changes in diet composition and their potential impact

Apart from the energy systems used, a more likely reason for the inconsistency in outcomes between the present study and the cited studies mentioned above is the methods by which the dietary energy densities were adjusted. In Attia and Hassan (2017) and Mao et al. (2024) energy densities were adjusted by altering inclusions of vegetable oils, mainly soy oil. In the present experiment, millrun was included at 109 g/kg in combination with a small alteration of dietary crude protein to decrease the dietary NE density, which is clearly a radically different approach. In the present experiment, the 1.286 FCR of broilers exposed to heat stress and offered reduced NE diets matched that of broilers receiving standard NE diets under thermo-neutral conditions. While speculative, the likelihood is that the 109 g/kg millrun inclusion in the reduced NE diets, rather than the NE reduction *per se,* was responsible for the FCR improvement. Millrun typically contains 89 g/kg crude fiber, 136 g/kg acid detergent fiber and 344 g/kg neutral acid detergent fiber (Stas et al., 2024). Indeed, millrun in the present study was analysed to contain 182 g/kg CP, 55 g/kg fat and 394 g/kg of NDF. Mateos et al. (2012) suggested that the response to fiber inclusion in diets for broiler chickens depends on the source and level of dietary fiber and the characteristics of the diet and the physiological status of the bird. However, they concluded that the dietary inclusions of moderate amounts of coarse, insoluble fiber sources, such as oat hulls, at levels between 20 and 30 g/kg usually improves the growth performance of broilers fed low-fiber diets. The dietary inclusion of 50 g/kg oat hulls has been shown to increase relative gizzard weight by 90.5% (14.1 versus 7.4 g/kg), increase digesta content from 26.1 to 34.6% of the full organ and reduce gizzard pH from 3.25 to 2.08 to significant extents. This increase in gizzard weight and, presumably, functionality would be expected to enhance FCR. Similarly, Mateos et al. (2012) the dietary inclusion of 30 g/kg oat hulls significantly improved FCR by 2.92% (1.33 versus 1.37). Regrettably, gizzard-related measurements and millrun particle size were not quantified in the present study and should be considered in future research.

Another possible explanation for the improved feed efficiency observed in birds offered the reduced-NE diet is the small reduction in dietary crude protein, which was accompanied by increased inclusion of non-bound amino acids (NBAA). Notably, dietary crude protein was not constrained during formulation in order to enable least-cost formulation based on NE in the present study. One of the advantages of adopting an NE system is its compatibility with reduced-protein diets, in which NBAA can partially replace intact protein sources, such as soybean meal, while still meeting amino acid requirements of broilers (Chrystal et al., 2020). Reduced dietary crude protein has been reported to decrease water intake and water excretion under thermoneutral conditions, potentially due to lower metabolic costs associated with improved protein digestion and utlisation. Such effects may be particularly advantageous under heat-stress conditions. Furthermore, unlike amino acids derived from intact proteins, NBAA do not require enzymatic digestion and are immediately available for absorption and utilization. This, together with the potential enhancement of gastrointestinal function associated with the higher inclusion of millrun, may have contributed to the improved feed efficiency observed in birds fed the reduced-NE diet.

### Starch and protein disappearance rate ratios and performance

It is noteworthy that reducing dietary net energy significantly enhanced breast-meat yields by 11.3% as a main effect, which could be indicative of enhanced protein deposition. These outcomes are aligned with the Moughan (2003) assertion that efficient net protein synthesis is dependent upon the appropriate availabilities of amino acids and non-amino acid energy sources, predominantly glucose, at sites of protein synthesis. It follows that starch-protein disappearance rate ratios provide an indication of the balance between glucose and amino acids at sites of protein synthesis to generate protein deposition in skeletal muscles and growth (Wang et al., 2026). However, the negative linear relationship (r = −0.216; P = 0.197) between ileal starch to protein disappearance rate ratios and breast-meat yields was not significant in the present study.

Mathematically, relationships of starch or protein (N) disappearance rates with weight gain or FCR will be confounded by feed intakes; however, the calculation of starch:protein disappearance rate ratios eliminates this potentially confounding factor. It is then noteworthy that the starch-protein disappearance rate ratio in the distal ileum tended to be negatively related to weight gain (r = −0.306; P = 0.055). This may indicate that narrowing starch-protein disappearance rate ratios advantaged growth performance across this study. The relevance of starch-protein disappearance rate ratios to broiler performance was declared in Sydenham et al. (2017), where proximal jejunal starch-protein disappearance rate ratios were quadratically related to weight gain (r = 0.849; P < 0.001) and FCR (r = 0.838; P < 0.001) and a ratio of 3.59:1 would generate the maximum weight gain of 1265 g/bird ratio and a similar ratio of 3.88:1 would support the minimum FCR of 1.287 from 15 to 28 days post-hatch.

## Conclusion

As expected, heat stress significantly depressed feed intake, weight gain, and feed efficiency regardless of dietary NE density. An intended reduction in formulated NE density improved feed efficiency and breast meat yield in broiler chickens under both thermoneutral and cyclic heat stress conditions. Because the reduction in NE was achieved through concurrent changes in diet composition, including increased millrun inclusion and reduced crude protein concentration, the observed responses cannot be attributed to NE density alone. These dietary modifications are inherent to practical NE-based feed formulation and highlight the potential importance of dietary fibre or non-starch polysaccharides in influencing broiler performance and carcass characteristics.

## Authorship contribution statement

Carria Xie led the investigation, data collection, data analysis and manuscript preparation, including drafting and revising the manuscript. Peter H. Selle contributed to the study investigation, data analysis and manuscript drafting. Shemil P. Macelline was involved in study supervision, investigation, data analysis and manuscript revision. Mushin Al Anas and Eunjoo Kim contributed to animal feeding study, experimental design and manuscript revision. Mehdi Toghyani and Reza Barekatain contributed to the study methodology, supervision and manuscript revision. Sonia Y. Liu conceived the study, secured funding and resources, managed the project, and contributed to manuscript revision. All authors reviewed and approved the final manuscript.

## Disclosures

The authors declare that there are not any conflicts of interest.
